# African migrant men’s experiences and preferences for formal mental health help-seeking: meta-synthesis and recommendations

**DOI:** 10.1080/00049530.2024.2347639

**Published:** 2024-05-13

**Authors:** Faduma Abdikadir, Hannah V. Freeman, Natasha van Antwerpen, Melissa Opozda, Deborah Turnbull

**Affiliations:** a School of Psychology, University of Adelaide, Adelaide, Australia; b Freemasons Centre for Male Health and Wellbeing, South Australian Health and Medical Research Institute and University of Adelaide, Adelaide, Australia; c College of Medicine and Public Health, Flinders University, Darwin, Australia

**Keywords:** Africa, help-seeking, men, mental health, migrant, qualitative

## Abstract

**Objective:**

Despite high rates of mental illness and significant barriers to accessing assistance, little is known about African migrant men’s views on formal mental health help-seeking (i.e. from a health professional) in their new countries. We aimed to synthesise qualitative literature on African migrant men’s experiences and preferences regarding formal mental health help-seeking in their new countries, and develop recommendations from the findings.

**Method:**

Systematic searches of six databases (nil date restrictions) for qualitative data from adult men who had migrated from any of the 16 countries in Africa with largest numbers of emigrants to any country outside of Africa, for any reason. Study quality was assessed using the Qualsyst tool with a minimum .55 total for inclusion. Extracted data were synthesised using meta-aggregation.

**Results:**

Five high quality studies (Qualsyst totals .80+) met inclusion criteria. All men had migrated to “Western” countries. One synthesised finding on help-seeking barriers was generated: African migrant men do not seek formal mental health help due to stigma and discrimination, a preference for religious treatment, structural barriers, and a perceived lack of cultural competency from health professionals.

**Conclusions:**

Recommendations are presented. Further research and co-design will be important to understand facilitators and develop culturally appropriate, accessible assistance.

## Key Points

**What is already known about this topic:**

1. Emigrants from African countries have very high rates of mental ill-health including anxiety, depression, and post-traumatic stress disorder.
2. African migrant men may hold cultural beliefs about how men should behave and mental illness that act as barriers to formal mental health help-seeking in their new countries.
3. Despite their potentially unique needs, existing data on African migrant men’s own views of formal mental health help-seeking have not been synthesised.

**What this topic adds:**

1. This is the first meta-synthesis of African migrant men’s experiences and beliefs related to formal mental health help-seeking in their new countries.
2. Our study synthesises data from five studies to describe barriers to help-seeking in this population.
3. Based on the findings of this study, we make a series of practical recommendations to reduce barriers and facilitate help-seeking in this population. The limited number of studies also highlights the need for further research on this topic.

African migration has steadily increased over the past two decades. In 2020, 21 million Africans were living in another country within Africa, and a further 20 million were living outside Africa (International Organisation for Migration, 2021). After another African country (53%), Europe (26%) is the most common destination for migrants, followed by Asia (11%), and North America (8%; International Organisation for Migration, 2021). Africa is a massive, complex, and highly diverse continent and individuals emigrate elsewhere for a wide variety of reasons. Despite stereotypes of mass African migration driven by poverty, war, famine, and underdevelopment, recent research indicates that much recent African emigration is instead related to development and social change resulting in increased desires to utilise capabilities and achieve ambitions (Flahaux & De Haas, 2016).

Regardless of the reason or level of preparation, the stressors associated with migration to a new country and often a new culture can have a profound impact on migrants’ mental health and wellbeing (Virupaksha et al., 2014; Ward et al., 2005). Migrating due to war, human rights violations, or financial hardship can particularly contribute to psychological vulnerability (United Nations Refugee Agency, 2016). African migrants may suffer multiple stressors in their new country including the continued effects of trauma, involuntary separation from family and friends, the need to learn a new language, cultural, gender, and role differences in their new country, lack of information and support, difficulty accessing vital services, unstable income, discrimination and racism, loss of cultural and religious rituals, and decreased connectedness (Abur & Spaaij, 2016; Kuyini & Kivunja, 2018; Mwanri et al., 2022; Pittaway et al., 2009; Savic et al., 2013). Overall, one in every eight people worldwide (12.5%) lives with a mental health condition (World Health Organisation, 2022), though the 2020 World Migration Report (International Organisation for Migration, 2021) notes higher rates of distress and mental health issues including stress, depression, anxiety, and post-traumatic stress disorders (PTSD) in migrants than in native-born populations and a recent meta-analysis (1091 articles; *N* = 28,367) found very high rates of anxiety (35%), depression (33%), and PTSD (38%) specifically among African migrants (James et al., 2022).

Within this population, the mental health treatment-related beliefs and preferences of African migrant men is of particular interest. In 2020, there were 22 million African male migrants; almost double the number in 2000 (12 million; United Nations, 2020). In African cultures characterised by traditional ideologies and masculine beliefs, men are viewed as important, intelligent, strong, and responsible for their families (Odimegwu & Okemgbo, 2008). Mental health receives little government and policy attention and is highly stigmatised in many parts of Africa (Ezeugwu & Ojedokun, 2020). Further, in some regions mental illness is commonly viewed as a supernatural or predominantly religious issue, rather than a medical condition (Kyei et al., 2014; Patel & Stein, 2015). With mental illness so stigmatised and prevalent masculine ideals that men should be strong, resilient, caretakers, African men may only be willing to seek “formal” help (i.e., from a health professional such as a medical doctor or psychologist) after supernatural and/or religious explanations and assistance have failed and their mental health has worsened or become chronic (Ezeugwu & Ojedokun, 2020), or they may not be willing to access it at all (Olanrewaju et al., 2019). Overall, there is a large number of African migrant men living in non-African countries, high rates of mental illness among African migrants, and potential misalignment between traditional and religious explanations for mental illness and the biomedical explanations for mental illness that are dominant in many countries that African men migrate to. Accordingly, there is a need to understand African migrant men’s perspectives of mental illness and its treatment to be able to provide services that are effective and culturally appropriate for this growing community.

A review of existing qualitative data is appropriate for this research topic. Qualitative research aims to generate an in-depth understanding of attitudes, beliefs, and experiences, and qualitative syntheses of findings from multiple qualitative studies are widely recognised as a valuable tool to facilitate an understanding of experiences and perspectives across a wider range of populations and/or contexts than would normally occur within a single study. Qualitative syntheses are particularly useful for identifying research gaps and informing the development, implementation, and evaluation of health interventions (Tong et al., 2012).

## Note on terminology and context

We use the term “migrant” throughout this paper to describe all categories of individuals who have left their home country to live in another. We note that migration may occur for many reasons, including as a voluntary decision (these individuals are commonly called migrants) or fleeing as a result of war, persecution, or human rights violations (these individuals are often referred to as asylum seekers or refugees, depending on their protection claim status; United Nations Refugee Agency, 2016). We also recognise that Africa is one of the most diverse continents in the world in aspects including culture, ethnicity, language, environment, infrastructure, government, conflict, politics, religion, social norms, and socioeconomic and development statuses (Appiah et al., 2018). In synthesising data from a range of men from various African nations, we acknowledge that these findings, while summarising the current qualitative literature on African migrant men’s perspectives on mental health help-seeking, may be reductive of the diversity of experiences and viewpoints seen in men across the continent.

## Aims

This meta-synthesis aims to:

1. Identify, appraise, and synthesise qualitative findings from studies exploring African migrant men’s preferences for and experiences of formal mental health help-seeking.
2. Use the synthesised findings to develop recommendations to facilitate African migrant men’s access to formal mental health services.

## Materials and methods

### Study design

We conducted a systematic review and meta-synthesis based on a pre-registered PROSPERO protocol (CRD42022336513).

### Eligibility criteria

The search strategy was pre-defined and adapted for each database. The PICOS criteria are outlined below.

#### Population

We included studies that reported men’s experiences or perspectives about formal mental health help-seeking from a physical or mental health practitioner. We included studies on the most common mental health difficulties faced by migrants: general mental ill-health, depression, anxiety, and PTSD (Tomasi et al., 2022). Studies on pre-migratory mental health difficulties, neurological disorders, or developmental disorders were not includable. We sought data from adult (18+ years) men and/or males who had emigrated from any of the 16 African countries with the highest reported numbers of emigrants, as identified by the Africa Centre for Strategic Studies (2021): Algeria, Burkina Faso, Côte d’Ivoire, Democratic Republic of Congo (DRC), Egypt, Ethiopia, Ghana, Mali, Morocco, Nigeria, Somalia, South Africa, South Sudan, Sudan, Tunisia, and Zimbabwe. Participants migrating from other countries were not includable. We sought data on individuals who had migrated from any eligible African country to anywhere outside of the African continent. We used studies that included both male/men and female/women participants only if male/men findings were extractable.

#### Interventions/Exposures

Intervention studies were not includable.

#### Comparison

Studies with any or no comparator were eligible.

#### Outcomes

The outcomes were to identify and assess the literature on African migrant men’s experiences of and beliefs about formal mental health help-seeking, in order to develop recommendations for improving African migrant men’s access to formal mental health assistance.

#### Study/Research type

We included peer-reviewed, English-language studies that reported qualitative data – that is, data obtained through qualitative means (e.g., interviews) and analysed using qualitative methods (e.g., thematic analysis). Mixed methods studies were includable if qualitative data were reported separately and were extractable. Studies that were not peer-reviewed were excluded.

### Data sources and search strategy

We followed the Preferred Reporting Items for Systematic Reviews and Meta-Analyses (PRISMA) guidelines and Enhancing Transparency in Reporting the Synthesis of Qualitative Research (ENTREQ) statement (Supplementary Table S1) in this research (Page et al., 2021; Tong et al., 2012). We conducted comprehensive, systematic searches in PubMed, Scopus, Psych INFO, CINAHL, Embase, and Web of Science. No date restrictions were used in the original searches (conducted 5 June 2022) and secondary identical searches were conducted to update the original searches (i.e., between 5 June 2022 and 3 January 2023). Search strategies used Boolean logic and incorporated keywords related to mental health, help-seeking, migrants, men and qualitative research (Supplementary Table S2). Search strategies were adapted for each database and an experienced research librarian was consulted to optimise search approaches.

### Screening and review

The search results were imported into Covidence (Veritas Health Innovation, n.d.) and automatically deduplicated prior to title and abstract screening, full-text review, data extraction, and quality appraisals. FA screened all abstracts and reviewed all full-text papers, with 10% at each stage co-screened by DT or MJO. Any disagreements were resolved via discussion.

### Data extraction

A data extraction form was developed to collect data on fields, including study aim, study design, and number and characteristics of male participants. Themes identified by primary authors were extracted verbatim, with quotes to support each.

### Quality assessment

Studies that met inclusion criteria were assessed for methodological reporting quality using the Qualyst checklist for qualitative studies (Kmet et al., 2004). Each study was assessed on whether it met each of 10 quality criteria (Yes = 2, Partial = 1, No = 0) and a total was calculated by adding all ratings, and then dividing by the possible total. We prespecified a “liberal” total cut-off score of .55 for inclusion (Kmet et al., 2004). All studies were appraised by both FA and MJO with disagreements resolved through discussion.

### Meta-synthesis strategy

Data were synthesised using the JBI meta-aggregative approach, which collates qualitative evidence without re-interpreting the findings (Hannes & Lockwood, 2011). Meta-aggregation is particularly useful in healthcare research because it seeks to generate specific “lines of action” or recommendations to improve health policy and/or practice (Lockwood et al., 2020). “Findings” (themes) reported by the original authors were extracted, with supporting quotes. Those findings were then grouped based on their similarities and labelled with a corresponding title. The groups were then aggregated again based on meaning similarity to form “synthesised findings” (an overarching description of a collection of classified findings; Lockwood et al., 2020).

## Results

### Study selection

**Figure 1.**
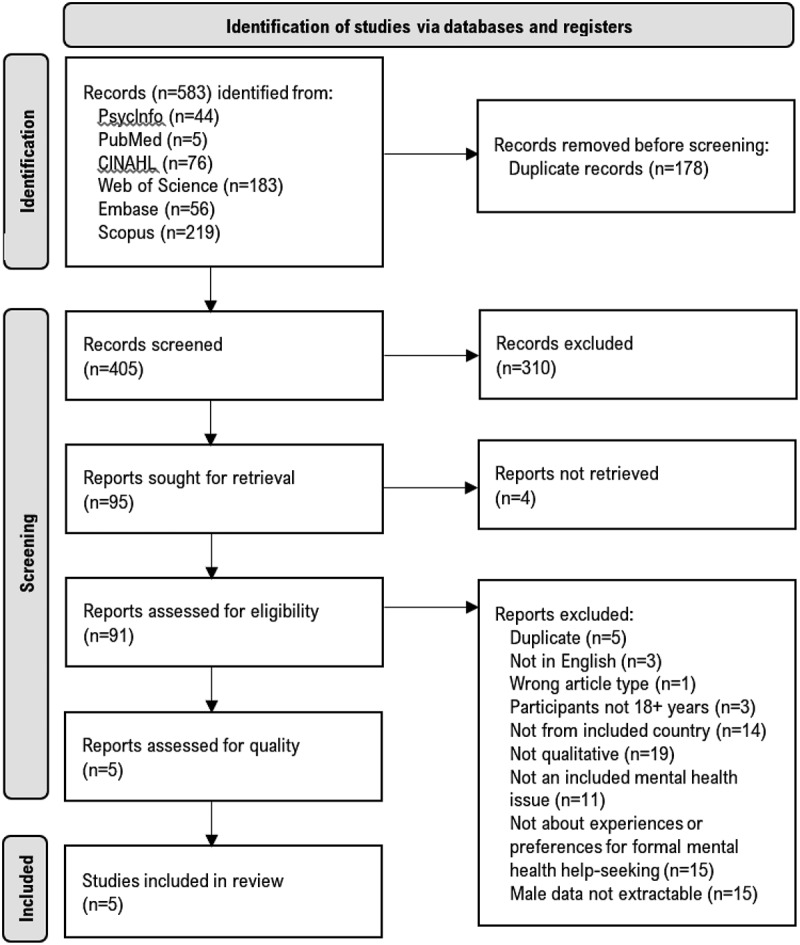
PRISMA 2020 flowchart.

Through database searches, we identified 405 non-duplicate records (Figure 1). Of these, 310 were excluded at title and abstract screening. A total of 91 records were then retrieved for full-text assessment, and 86 were excluded (see Figure 1 for exclusion reasons). Five eligible studies then moved on for quality appraisal. Inter-rater agreement was moderate between FA and DT (70%) at title and abstract screening and high between FA and MJO (96%) at full-text review.

### Quality assessment

**Figure 2.**
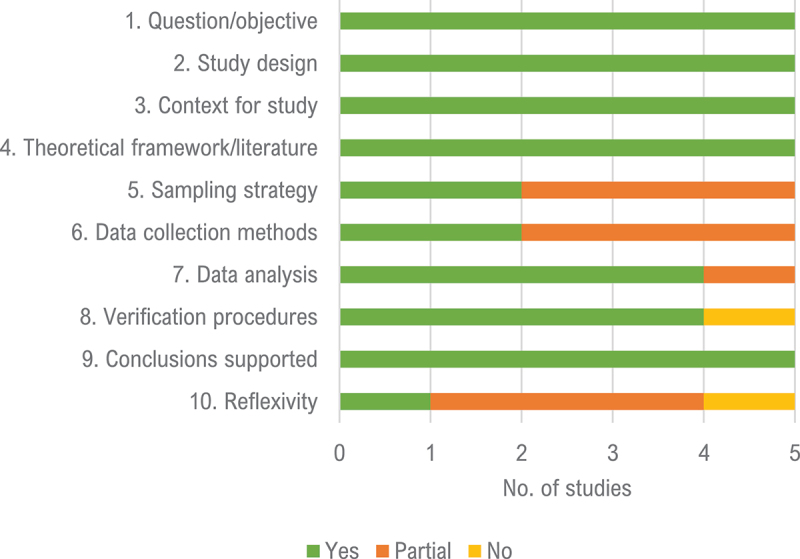
Aggregated study quality assessment item ratings.

Table 1 presents the total Qualsyst score for each assessed study (for a detailed assessment of each study, refer to Supplementary Table S3). Overall quality was high (lowest total of .80); as such, no paper was excluded for subthreshold quality score. As shown in Figure 2, all studies included a clear description of the question or objective, had an easily identified design that was appropriate to address the study question, provided a clear context for the study, included a description and justification of the theoretical framework or wider body of knowledge informing the study, and reported sufficient original evidence to support the conclusions. Four studies lacked reflexivity (an explicit assessment of the likely impact of the researchers’ personal characteristics and methods used on the data and results; Fauk et al., 2022; Grupp et al., 2019; Lechner-Meichsner & Comtesse, 2022; Mölsä et al., 2010), three did not completely describe or justify their sampling strategies (Fauk et al., 2022; Michlig et al., 2022; Mölsä et al., 2010) or did not clearly describe their data collection methods (Fauk et al., 2022; Grupp et al., 2019; Michlig et al., 2022), one study did not fully describe their analysis methods (Mölsä et al., 2010), and one did not report having used any verification procedures to establish the credibility or trustworthiness of the data (Mölsä et al., 2010).

**Table 1.** Study and male participant characteristics.

Author (year), country	Study characteristics				Male participants			Total quality score
	Aim	Recruitment	Qualitative data collection	Qualitative data analysis	n (% of total N)	Mean age, years (SD)	Included countries of origin	
Fauk et al. (2022), Australia	Explore personal, structural, sociocultural, and religious barriers to accessing mental health services among African migrants	African communities and migrant service organisations in South Australia were provided with study information	Individual interviews	Thematic analysis	10 (50%)	N/R for male participants	DRC, South Sudan, Ethiopia, Somalia	.85
Grupp et al. (2019), Germany	Understand help-seeking intentions and lay beliefs about cures for PTSD by asylum seekers from Sub-Saharan African countries living in Germany	Networked at cultural gatherings, and publicly approached potential participants; participants had participated in part 1 of the study and then consented to take part in a focus group	Focus groups (including 3 with participants from Somalia, 2 with participants from Cameroon)	Interpretative phenomenological analysis	24 (92%)	N/R for male participants	Somalia, Cameroon	.85
Lechner-Meichsner and Comtesse (2022), Germany	Identify illness beliefs and treatment expectations regarding prolonged grief disorder among Arab and sub-Saharan refugees	Convenience sample recruited via social media, word-of-mouth, and printed ads “in places frequented by refugees”	Individual semi-structured interviews	Qualitative content analysis	10 (44%)	N/R for male participants	Côte d’Ivoire, DRC, Ethiopia, Somalia	.90
Michlig et al. (2022), United States of America	Understand specific resistances to US mental health care contributing to the gap in which primary care is low among Somalis compared to other refugee groups	“Four gender-matched Somali facilitators recruited participants under the supervision of a Somali research team member”	Community forums (divided by gender) incorporating semi-structured focus groups (separate groups for youths, middle aged, and older participants)	“Critical discourse analysis”	84 (50%)	N/R	Somalia	.90
Mölsä et al. (2010), Finland	Examine how conceptions, expressions and treatment of mental distress are changing among Somalis living in Finland	“Participants were recruited with the help of two community activists”	Focus groups (2 with Somali seniors, divided by gender) and individual interviews (2 with Islamic healers)	Data translated from Somali/Finnish to English, and “organised thematically”	7 (35%)	N/R for male participants	Somalia	.80

DRC, Democratic Republic of Congo; N/R, not reported; SD, standard deviation

### Study characteristics

The characteristics of each included study are presented in Table 1. The studies were published between 2010 and 2022 and were conducted in Australia (Fauk et al., 2022), Germany (Grupp et al., 2019; Lechner-Meichsner & Comtesse, 2022), United States (Michlig et al., 2022) and Finland (Mölsä et al., 2010). Data were collected through individual interviews (Fauk et al., 2022; Lechner-Meichsner & Comtesse, 2022; Mölsä et al., 2010), focus groups (Grupp et al., 2019; Michlig et al., 2022; Mölsä et al., 2010), and community forums (Michlig et al., 2022). Data were analysed using thematic (Fauk et al., 2022; Mölsä et al., 2010), interpretative phenomenological (Grupp et al., 2019), qualitative content (Lechner-Meichsner & Comtesse, 2022), and “theory-driven” (Michlig et al., 2022) analyses.

### Participant characteristics

Table 1 depicts characteristics of the total 134 male participants in the included studies. All studies included both male/men and female/women participants, with men comprising 35–71% of the study samples. Male participants had migrated from Cameroon, Côte d’Ivoire, DRC, Ethiopia, Somalia, or South Sudan, and were living in Australia, Finland, Germany, or the United States. Demographic data on male study participants were limited as most studies had collapsed this information across gender.

### Synthesised finding

Data from the five included studies (Fauk et al., 2022; Grupp et al., 2019; Lechner-Meichsner & Comtesse, 2022; Michlig et al., 2022; Mölsä et al., 2010) were synthesised to produce one finding:

*African migrant men do not seek formal mental health help in their new countries due to stigma and discrimination related to mental illness, a preference for religious and spiritual treatment, perceived lack of cultural competency among health professionals, and structural difficulties related to accessibility.*

The four categories underlying this synthesised finding are reported below.

#### Category 1: African migrant men do not seek formal mental health help due to perceived stigma and discrimination

African migrant men reported not seeking formal mental health help due to stigmatising labels and discrimination related to mental illness within their communities. They feared being socially isolated (DRC; Fauk et al., 2022), ostracised from their community (DRC; Lechner-Meichsner & Comtesse, 2022), being “mocked or looked down at” (DRC; Fauk et al., 2022, p. 3), or being called “crazy” and “possessed by demons” (Somalia; Michlig et al., 2022, p. 5). Somali migrant men noted that “madness [waalli]” was associated with “kill[ing] human beings or animals” (Mölsä et al., 2010, p. 284) and would lead to being cast out of their community: “You’re not a part of community anymore because you have a mental health issue. (…) You are considered an outcast” (DRC; Fauk et al., 2022, p. 10). One Somali man also noted that people may hide their mental illness to avoid the potential consequences of disclosure: “There are things that cannot be said out loud, like depression. [If you tell someone you are depressed], they will ask ‘are you mad?’ … [and say] you should be brought to the mental hospital” (Somalia; Michlig et al., 2022, p. 5).

#### Category 2: African migrant men who abide by traditional beliefs may preference religious and spiritual assistance over ‘western’ therapies

African migrant men from Ethiopia reported beliefs that mental illness is “God’s punishment” or caused by “supernatural… evil”. This belief was said to “hinder or stop people from seeking mental health services” (Fauk et al., 2022, p. 12). Similarly, men from Somalia stated that people experiencing mental illness are seen to be “affected by a curse (inkaar)”, the “evil eye”, or “afflicted by saar” (i.e., spirits; Mölsä et al., 2010, p. 285). These aetiological beliefs leads men to “trust more to the Church and the priest around them” (Ethiopia; Fauk et al., 2022, p. 12), with some believing that only religion can help in these situations: “Psychologists in Africa are the priests and the pastors … A psychologist cannot help a traditional person … If he is caught in this feeling, only older people who have grown up in this tradition can talk to him … ” (Ethiopia; Lechner-Meichsner & Comtesse, 2022, p. 13).

Religious leaders were said to offer treatments. Somali men described them in ways such as: “give (…) this small water (…) it’s called Taleeth [holy water]” and is said to “help inside” (Grupp et al., 2019, p. 9) and Cameroonian men noted the importance of traditional or spiritual healers called Marabouts: “A Marabout is like a doctor for the mind. A doctor of spirits” (Grupp et al., 2019, p. 10). Men from Cameroon also described treatments for PTSD symptoms and other mental illness involving sacrificial offerings that would be performed by the person’s family in their country of origin:

You can ask them to send money. With this money you can buy a goat or a chicken. And one will kill the chicken. Or to buy candies and do a Sadaqa (charity), share it in the neighbourhood or in the village so that he can receive the blessing. (Grupp et al., 2019, p. 10)

Some Somali men also reported prioritising religious treatment over that from mental health professionals in order to maintain connection with their traditional values and religious beliefs (Michlig et al., 2022).

#### Category 3: African migrant men are uncertain of mental health clinicians’ levels of cultural competency and understanding

Some African migrant men were reluctant to seek support due to perceived cultural differences between them and mental health professionals. These included different understandings of mental health, polarised upbringings, and uncertainty related to Western therapies. One Somali man said he did not want to seek help because he had heard from a friend that “they [the mental health professional] didn’t believe him … ” and that “they don’t understand what is this problem … ” (Grupp et al., 2019, p. 11), and because of concerns that a therapist would not be able to “digest” his situation because they would not have had the same experiences and would be unfamiliar with the client’s culture and traditions (Grupp et al., 2019, p. 11). Men also expressed concerns about potential communication difficulties in attempting to talk to a local mental health clinician:

It is very difficult for an adult to go to a doctor and have no means to express his feelings and pain. At the same time after you leave the reception you are never sure whether you understood the instructions of the doctor or if you might use the medicine incorrectly. (Somalia; Mölsä et al., 2010, p. 293)

Accordingly, Somali men suggested that there was a need to train people from their own culture as mental health professionals to facilitate culturally competent care:

For example, when I visited Minnesota, I went to a bank and there was a man who deals with the Somali people who don’t [speak English]. One of the reasons why people move out of this state [Arizona] is we don’t have that kind of service … We came here, and we will be here, so we need our own people to be trained as doctors. (Michlig et al., 2022, p. 6)

#### Category 4: African migrant men do not seek formal mental health help due to structural barriers such as location and accessibility

Men also identified practical barriers that prevent them from seeking formal mental health help in their new country. Financial dependency, location and limited availability were particularly highlighted (Fauk et al., 2022; Lechner-Meichsner & Comtesse, 2022). It was noted that many “refugees now tend to stay in groups in certain areas” (South Sudan; Fauk et al., 2022, p. 8), and when services were not available close to that area, access was often difficult: “Not every family has access to a vehicle and when it comes to such thinking: we’ll catch one bus and then we’ll go and catch another one and that becomes a bit of problem” (DRC; Fauk et al., 2022, p. 8). Men from the DRC who had migrated to Australia also identified limitations of hospital emergency departments as a barrier to accessing mental health help, including “waiting for a long time (…) to see doctors” and that “it is not actually immediate service which you can get straight away” (Fauk et al., 2022, p. 9). In contrast, Somali men living in Finland appreciated the country’s health service “advanced technology and high-standard research” (Mölsä et al., 2010, p. 292).

## Discussion

High rates of African emigration, alongside concerns of mental illness among migrant populations, make it important to understand perspectives and attitudes around help-seeking for mental illness in migrants’ new countries. Understanding the perspectives of African migrant men is particularly important, given potential interactions between cultural beliefs about how men should behave and the dominant medical models of mental health in the countries to which they often migrate. We synthesised qualitative data from African migrant men on their beliefs about seeking formal mental health assistance in their new countries to identify ways to develop culturally-appropriate, acceptable mental health assistance for this population. We identified five high-quality studies in which male participants had migrated from one of six African countries (Cameroon, Côte d’Ivoire, DRC, Ethiopia, Somalia, South Sudan) to one of four “Western” countries (Australia, Finland, Germany, United States). Data were synthesised into one finding comprising four barriers to African migrant men seeking formal mental health help in their new countries:

1. Stigma and discrimination related to mental illness.
2. Preferences for religious and spiritual treatment.
3. Perceived lack of cultural competency among health professionals.
4. Structural difficulties related to location, transport, and cost.

These findings align with those from other qualitative studies with comparable samples. Selected examples include accounts of Somali-Australian women who identified stigma, mistrust of Western healthcare systems, and concerns about cultural disconnect as barriers to help-seeking (Said et al., 2021). Norwegian research with migrants from Somalia, Eritrea, and Sudan who had sought help for substance use problems also points to similar barriers including scepticism towards a “white system” and fear of exclusion by their family and ethnic community (Pettersen & Debesay, 2023). Research with undocumented migrants to the Netherlands including men and women from various African countries also identified a preference for religious support first, as well as barriers including taboos related to mental health problems and structural difficulties such as financial and practical barriers (Teunissen et al., 2014). Our findings, considered alongside previous research such as this, point to the need for a biopsychosocial approach to mental health care provision for such migrant groups, to take into account the wide range of influences and context of lived experiences of those requiring mental health support (Kronick, 2018).

Table 2 displays recommendations for facilitating formal mental health help-seeking by African migrant men arising from the results of this research. Our study suggests that further work in a number of areas will be helpful to reduce barriers and develop ways to facilitate African migrant men’s formal mental health help-seeking in their new countries. Overall, our recommendations suggest three main areas for consideration: improving mental health literacy among African migrant men and their communities, collaboration between African communities and psychologists to provide culturally competent care with consideration for religious beliefs, and improving African migrant men’s ability to access services. While our recommendations make references to existing Australian services, we note that these types of services may not exist in other countries – conversely, other countries may already have programmes and services that effectively meet the needs of African migrant men to enable their formal mental health help-seeking. Our recommendations are designed to be considered and adapted according to local situations and existing services.

**Table 2.** Recommendations to facilitate formal mental health help-seeking by African migrant men.

Barrier(s)	Recommendation	Practical Implementation Guide
Stigma and discrimination related to mental illness	Improve mental health literacy in African migrant communities through access to culturally appropriate education and information	Co-design culturally sensitive, appropriate mental health literacy education programmes. Implement and/or bolster education programmes with current information on aeitology, symptoms and treatment options for psychological difficulties into African migrant communities in Australia. Leverage existing sources of information to communicate mental health knowledge, services, and access, e.g., communicating through the humanitarian off-shore programme or via employers of skilled migrants (Forshaw, 2011). Be mindful of the use of language and terminology that may be stigmatising, e.g., “mental illness”, and consider using less stigmatising language, e.g., “wellbeing” (Fauk et al., 2022).
Preferencing religious or traditional treatment over Western therapies	Improve the intergration of African migrant community members with mental health services	Develop collaborations between African migrant community leaders and religious healers (e.g., priests) with Australian mental health professionals to co-design and communicate culturally appropriate services. Consider opportunities to train community members as mental health care providers or lived experience advocates.
Uncertainty about mental health clinicians’ cultural competency and understanding	Mental health professionals to collaborate with community leaders and religious healers to develop trust alongside culturally appropriate services and understanding	Collaboration might build on previous programmes (e.g., the Beyond Blue’s NewAccess and NewRoots in Australia), and be facilitated and promoted through professional psychological and medical organisations. Build education on the impact of African cultural beliefs on mental health help-seeking into university curricula and continuous professional development schemes.
Structural barriers such as location, distance, and cost	Improve accessibility of services for African migrant men through free and anonymous digital mental health tools, telehealth, and location of mental health services	Researchers, mental health organisations, multicultural service providers, and African migrant men should co-design culturally appropriate digital mental health services. Availability of mental health assistance via telehealth should be promoted, where available, to African migrant communities. Increase accessibility of clinics through location near African migrant communities and/or provide African migrant men with education on accessing clinics via public transport.

Contrasting with the amount of data we found on barriers to help-seeking, we identified very little data on help-seeking facilitators. The few data we saw suggested that positive experiences with mental health professionals (Lechner-Meichsner & Comtesse, 2022), having community members advocate for formal help-seeking (Michlig et al., 2022), and the availability of culturally competent mental health professionals (Fauk et al., 2022) may enable African men’s formal mental help-seeking. Supporting these ideas, a recent mixed-gender interview study of African migrants and service providers in South Australia also found that that promotion of mental health services by community members and leaders and culturally competent mental health services would facilitate formal help-seeking (Fauk et al., 2022).

Education to promote mental health literacy, including an understanding of mental illnesses and normalisation of mental health services from arrival to Australia, may assist in combating the stigma that African migrant men report as being a barrier to accessing assistance. The importance of improving mental health literacy and awareness of the benefits of service use among African migrant populations has long been highlighted in the literature (Jorm et al., 2006), with our findings suggesting need for this work persists. As our findings suggest that men’s concerns of stigma are primarily related to perceptions of others in their community, such efforts should also ensure that information to promote better mental health literacy is also provided to wider communities rather than just targeting African migrant men. Such education may also benefit from use of less stigmatised terminology, such as using “wellbeing” or culturally appropriate terms identified by specific communities, in place of terms such as “mental health”, “mental illness”, or “psychology” (Fauk et al., 2022).

Our study also found that religious and spiritual values may play important roles in shaping African migrant men’s mental health help-seeking behaviour. Men’s preferences for religious treatment and their perceptions of poor quality of cultural competence amongst mental healthcare professionals suggest a need for greater collaboration between Australian mental health care professionals and community leaders, including religious leaders. Such collaboration could be mutually beneficial – providing community leaders with awareness on mental health and available services, and providing psychologists with an understanding of African cultural beliefs that may impact on appropriate therapeutic practices. In Australia, the Transcultural Mental Health Centre provides anonymous, telephone-based, culturally competent assistance to help migrants access mental health care, by connecting callers “to experienced clinicians who can speak their language and who understand the person’s culture”. They offer callers advice on mental health self-management strategies, help to access services in their own community, and provide support for carers (Transcultural Mental Health Centre, 2023). However, further efforts appear to be needed to develop culturally competent mental health professionals, and it is unclear whether any “Western” mental health services have sought to work with or alongside African men’s often preferred avenue for mental health assistance – religious and spiritual healers.

The related concepts of cultural competence and cultural humility play key roles in ensuring that equitable services are provided for clients from diverse backgrounds and that clients feel comfortable with healthcare professionals, leading them to continue to seek and use professional services (Bennegadi, 2009; Lekas et al., 2020). The perception that clinicians lack the skills to effectively work with African migrant men suggests a need to provide training and resources to improve clinicians’ abilities to effectively work with and assist people experiencing mental ill-health in this population. Such training should include the development of (1) knowledge: in sociocultural factors impacting mental health and beliefs, values, and behaviours related to mental health, (2) abilities: including in intercultural communication and diagnosis in diverse populations, and (3) attitudes: including an understanding of the influence of culture on beliefs and values, and respect for differences even where they conflict with personal values (Bennegadi, 2009). Alongside these important competencies, specific development of “cultural humility” will also be key. In this concept, practitioners are encouraged to reflect on and challenge their own beliefs and biases, admit what they do not know and be willing to learn from their client, and to cultivate respectful, person-centred caring that is right for the individual (Lekas et al., 2020). While the field is increasingly moving towards a focus on cultural humility rather than cultural competence, training in both may be more effective than cultural humility alone, given participant concerns that practitioners do not understand important aspects of their culture. Cultural training should therefore incorporate general resources and training in cultural humility, alongside specific resources for different population groups – including African migrant men. Cultural competence and humility in this context might be developed within both formal academic training, and professional development and networks to ensure ongoing and up-to-date information (Bennegadi, 2009).

Co-design is likely to be beneficial for developing and implementing mental health services that will be acceptable and effective for African migrant men. Co-design moves away from programme design that is driven by experts, to active collaboration with potential programme users across the entire research process, with users acting as experts in their preferences and needs (Slattery et al., 2020; Visser et al., 2005). Such co-design could also assist the formation of gender-sensitive health promotion interventions (those designed specifically for the circumstances, needs, and wants of men) which are often more effective for men than non-gender-sensitive interventions (Robertson & Baker, 2017; Seaton et al., 2017). Combining co-design with appreciative inquiry, which takes a strengths-based approach, could be beneficial in this work in order to encourage a focus on what is or could be going well as well as self-directed change at the individual and institutional levels (Ghosh et al., 2022). Research to gather further data on facilitators to African men’s mental health help-seeking would also be helpful in implementing co-design and appreciative inquiry approaches that go beyond just addressing barriers (Merriel et al., 2022).

African migrant men also reported structural barriers to accessing formal mental health help, such as clinics being located far from their communities, high cost of services, and difficulty accessing transport to appointments. While the most obvious answer to this might appear to be to locate culturally appropriate services in areas where African migrants reside, that is not always possible or practical. Community programmes aimed at practical skills such as using public transport might also assist African migrant men to be able to reach appointments. Alternate means of providing acceptable services that overcome these barriers may be in the use of digital services, such as mental health assistance provided by clinicians via telehealth or via co-development of digital mental health tools (e.g., apps or websites) that provide culturally competent, accessible mental health education and therapeutic strategies for African migrant men. Digital mental health assistance may also overcome concerns related to stigma and privacy reported by African migrant men as barriers to help-seeking. However, use of digital mental health services requires digital access and literacy. Digital inclusion is relatively high across culturally and linguistically diverse populations in Australia, except among newly arrived migrants, who often experience digital exclusion. As such, while digital mental health strategies may often be more appropriate for established migrant populations, such strategies should be rolled out alongside those aiming to improve digital access and digital literacy in both newly arrived and more established migrant populations. This may have benefits for wellbeing via social connection, employment, education, information, and services (Settlement Council of Australia, 2020; Tour et al., 2023).

Our pragmatic, pre-determined decisions to only include studies that (a) discussed the mental illnesses most common to African migrants and general mental unwellness, and (b) included data from male migrants only from the most common African emigration countries, resulted in only five included studies. As we only sought peer-reviewed papers, some relevant data may also have been missed in grey literature and reports. However, our approach ensured a high-quality sample on a specific population of interest, and the low number of studies highlights the need for ongoing work in this area. As acknowledged earlier, our synthesis does not cover the full scope of African regions and cultures, and also presents findings from men living in four destination countries. However, we note that past research suggests some indication of a “united voice” across African migrant cultures (Fauk et al., 2022) with some common issues such as stigma in help-seeking seen across destination countries (Knipscheer & Kleber, 2008; Mantovani et al., 2017). We hope that our analysis of the available literature provides a helpful starting point to understand findings on this topic and that research will become more nuanced as work in this area continues. Future work may also benefit from considering mental health in the context of the healthy immigrant effect (wherein new migrants are commonly healthier than native-born populations, an effect which usually vanishes with longer residency; Elshahat et al., 2022), duration of residence, and reason for migration. However, as findings on the healthy immigrant effect suggest it may be more relevant to physical health than mental health outcomes, a more nuanced account of factors related to post-migration health and wellbeing such as language proficiency, experiencing forced migration, and experiences of racism and discrimination in their new country are likely to be more useful in considering and developing migrant mental health interventions (Elshahat et al., 2022; Lee, 2018). Accordingly, further research may look to include these factors when studying mental health and related outcomes in populations of African migrant men.

## Conclusion

This is the first meta-synthesis of African migrant men’s experiences and preferences of seeking formal mental health care in their new countries. Data synthesised from five studies on barriers to help-seeking in this population informed our core recommendations for: (1) education and communication to improve mental health literacy in African migrant populations, with consideration for the use of non-stigmatising language, (2) collaboration between psychologists and community and religious leaders to improve cultural awareness and appropriateness of services, and (3) development of culturally appropriate digital tools and programmes, and practical skills to address structural barriers to care access. In addition to these suggestions, future research would benefit from a focus on identifying facilitators of African migrant men’s formal help-seeking, and the use of co-design to produce acceptable, accessible, and culturally appropriate services.

## Supplementary Material

Supplemental Material

## Data Availability

Data sharing is not applicable as no new data were created or analysed in this study.
