# Supplementary material for: African migrant men’s experiences and preferences for formal mental health help-seeking: meta-synthesis and recommendations
Source: Aust J Psychol. 2024 May 13;76(1):2347639. doi: 10.1080/00049530.2024.2347639 (PMC12218550; doi:10.1080/00049530.2024.2347639)
Supplement: Supplemental Material [file RAUP_A_2347639_SM9997.docx]

**Supplementary Table 1. ENTREQ checklist**

| **Item** | **Guide and description** | **Page No.** |
| --- | --- | --- |
| Aim | Use synthesised findings to understand African migrant men’s experiences and preferences for formal mental health help-seeking. Develop recommendations for engaging African migrant men in formal mental health help-seeking. | 22 |
| Synthesis methodology | Meta-aggregation - an approach to qualitative evidence synthesis (QES) developed by the Joanna Briggs Institute (JBI) | 22 |
| Approach to searching | Comprehensive search strategies to seek all available studies | 23 |
| Inclusion criteria | Sample comprises of men who have migrated from the 16 identified African countries, studies published in English and had qualitative data, data on male were extractable, peer-reviewed, over 18 years of age | 23 |
| Data sources | To ensure comprehensive search, 6 electronic databases was used (PubMed, Scopus, PsycINFO, CINAHL, Embase and Wed of Science) | 23 |
| Electronic search strategy | See Supplementary Table 2 | 64 |
| Study screening methods | AF and DT/MJO screened 10% of the articles. | 24 |
| Study characteristics | See Table 1 | 29 |
| Study selection results | See Table 1 | 29 |
| Rationale for appraisal | The Qualsyst Quality Assessment Checklist was used as it a common appraisal tool for meta-synthesis | 25 |
| Appraisal items | Appraisal items included: Question/objective, design evident, context, theoretical framework, sampling strategy, data collection, data analysis, verification procedure, conclusion, reflexivity of the account | 31 |
| Appraisal process | Appraisal was conducted by the author and a supervisor; any disagreements was resolved through discussion with a second supervisor | 27 |
| Appraisal results | Appraisal scores ranged from 0.80 to 0.90, indicating the studies to be high quality, see Table 2 | 31 |
| Data extraction | All data are represented in Table 1 | 69 |
| Software | N/A | N/A |
| Number of reviewers | Two | N/A |
| Coding | The author read all five papers to identify themes and merged them together based on their similarities | 28 |
| Study comparison | Translating qualitative studies into one another | 28 |
| Derivation of themes | Derived the themes through the primary author’s interpretation and constant comparison | 28 |
| Quotations | See results section | 32 |
| Synthesis output | See results section | 32 |

**Supplementary Table 2. Search strategy for each database**

|  | **Mental health** | **Help seeking** | **Africa** | **Migrant** | **Men** | **Qualitative** |
| --- | --- | --- | --- | --- | --- | --- |
| **Pubmed** | ("mental health"[mh] OR anxiety[mh] OR "Stress Disorders, Post-Traumatic"[mh] OR "depression"[mh] OR "depressive disorder"[mh] OR "mental health" [tiab] OR "mental illness" [tiab] OR "mental disorder" [tiab] OR depress* [tiab] OR anxiety [tiab] OR "post traumatic stress disorder" [tiab] OR ptsd [tiab] OR trauma* [tiab] OR stress* [tiab]) | "Help-Seeking Behavior"[mh] OR help seek* [tiab] OR mental health help seek* [tiab] OR service use[tiab] OR seek advice [tiab] OR help* [tiab] OR service utilis*[tiab] OR service utiliz*[tiab] OR access*[tiab] | (Africa* [mh] OR Africa[tiab] OR Egypt* [tiab] OR Tunisia* [tiab] OR Algeria* [tiab] OR Morocc* [tiab] OR Ghan* [tiab] OR Mali* [tiab] OR Nigeria* [tiab] OR "Democratic Republic of Congo" [tiab] OR Ethiopia* [tiab] OR "Côte d’Ivoire" [tiab] OR "South Africa*" [tiab] OR "Burkina Faso" [tiab] OR Somali* [tiab] OR "South Sudan*" [tiab] OR Sudan* [tiab] OR Zimbabw* [tiab]) | ("refugees"[mh] OR "Transients and Migrants"[mh] OR migrant* [tiab] OR refugee* [tiab] OR asylum seeker* [tiab] OR immigrant* [tiab] OR settler* [tiab] OR newcomer* [tiab] OR displaced people[tiab] OR displaced person*[tiab]) | (men [mh] OR male* [tiab] OR man [tiab]) | ("qualitative research" [mh] OR qualitative [tiab] OR "focus groups" [mh] "focus group*" [tiab] OR interview* [tiab] OR narrative* [tiab] OR "discourse analys*" [tiab] OR "thematic analys*" [tiab], theme* [tiab] OR "content analys*" [tiab] OR "grounded theory*" [tiab] OR "lived experience*" [tiab] OR "personal experience*" [tiab] OR "mixed method*" [tiab]) |
| **CINAHL** | (MH "mental health" OR MH anxiety+ OR MH "anxiety disorders+" OR MH depression OR MH "Stress Disorders, Post-Traumatic+" OR TI "mental health" OR AB "mental health" OR TI "mental illness" OR AB “mental illness" OR TI "mental disorder" OR AB "mental disorder" OR TI depress* OR AB depress* OR TI anxiety OR AB anxiety OR TI "post traumatic stress disorder" OR AB "post traumatic stress disorder" OR TI ptsd OR AB ptsd OR TI trauma* OR AB trauma* OR TI stress* OR AB stress*) | (MH "Help Seeking Behavior" OR TI "help seek*" OR AB "help seek*" OR TI "mental health help seek*" OR AB "mental health help seek*" OR TI "service use" OR AB "service use" OR TI "seek advice" OR AB "seek advice" OR TI help OR AB help* OR TI "service utilis*" OR AB "service utilis*" OR TI "service utiliz*" OR AB "service utiliz*" OR TI access* OR AB access*) | (MH Africa+ OR TI Africa* OR AB Africa* OR TI Egypt* OR AB Egypt* OR TI Tunisia* OR AB Tunisia* OR TI Algeria* OR AB Algeria* OR TI Morocc* OR AB Morocc* OR TI Ghan* OR AB Ghan* OR TI Mali* OR AB Mali* OR TI Nigeria* OR AB Nigeria* OR TI "Democratic Republic of Congo" OR AB "Democratic Republic of Congo" OR TI Ethiopia* OR AB Ethiopia* OR TI "Côte d’Ivoire" OR AB "Côte d’Ivoire" OR TI "South Africa*" OR AB "South Africa*" OR TI "Burkina Faso" OR AB "Burkina Faso" OR TI Somali* OR AB Somali* OR TI "South Sudan*" OR AB "South Sudan*" OR TI Sudan* OR AB Sudan* OR TI Zimbabw* OR AB Zimbabw*) | (MH refugees OR MH "Transients and Migrants" OR TI migrant* OR AB migrant OR TI refugee* OR AB refugee* OR TI "asylum seeker*" OR AB "asylum seeker*" OR TI immigrant* OR AB immigrant* OR TI settler* OR AB settler*) | (MH men OR MH "men’s health" OR MH male OR TI men OR AB men OR TI men’s OR AB men’s OR TI male OR AB male) | (MH "qualitative studies+" OR MH focus groups OR MH interviews+ OR TI qualitative* OR AB qualitative* OR TI "focus group*" OR AB "focus group*" OR TI interview* OR AB interview* OR TI "discourse analysis" OR AB "discourse analysis" OR TI narrative OR AB narrative OR TI thematic OR AB thematic OR TI theme* OR AB theme* OR TI "grounded theory" OR AB "grounded theory" OR TI phenomenology OR AB phenomenology OR TI experience* OR AB experience*) |
| **Scopus** | TITLE-ABS("mental health" OR "mental illness" OR "mental disorder" OR depress* OR anxiety OR "post traumatic stress disorder" OR ptsd OR trauma* OR stress*) | TITLE-ABS("help seek*" OR "mental health help seek*" OR "service use" OR "seek advice" OR help OR "service utilis*" OR "service utiliz*" OR access*) | TITLE-ABS(Africa* OR Egypt* OR Tunisia* OR Algeria* OR Morocc* OR Ghan* OR Mali* OR Nigeria* OR "Democratic Republic of Congo" OR Ethiopia* OR "Côte d’Ivoire" OR "South Africa*" OR "Burkina Faso" OR Somali* OR “South Sudan*” OR Sudan* OR Zimbabw*) | TITLE-ABS(migrant* OR refugee* OR "asylum seeker*" OR immigrant* OR settler*) | TITLE-ABS(men* OR male* OR man*) | TITLE-ABS(qualitative* OR "qualitative research*" OR "focus group*" OR interview* OR narrative* OR "discourse analys*" OR "thematic analys*" OR "content analys*" OR "grounded theory*" OR "lived experience*" OR "personal experience*" OR "mixed methods*") |
| **Embase** | (mental health.sh OR mental disease.sh OR exp anxiety OR exp posttraumatic stress disorder OR exp depression OR depress*.ti,ab OR anxiety.ti,ab OR post-traumatic stress disorder.ti,ab OR ptsd.ti,ab OR trauma*.ti,ab OR stress*.ti,ab) | (help seeking behavior.sh OR help seek*.ti,ab OR mental health help seek*.ti,ab OR service use*.ti,ab OR seek advice.ti,ab OR help.ti,ab OR service utilis*.ti,ab OR service utiliz*.ti,ab OR access*.ti,ab) | (exp Africa OR Africa*.ti,ab OR Egypt*.ti,ab OR Tunisia*.ti,ab OR Algeria*.ti,ab OR Morocc*.ti,ab OR Ghan*.ti,ab OR Mali*.ti,ab OR Nigeria*.ti,ab OR Democratic Republic of Congo.ti,ab OR Ethiopia*.ti,ab OR "Cote d Ivoire".ti,ab OR South Africa*.ti,ab OR Burkina Faso.ti,ab OR Somali*.ti,ab OR South Sudan*.ti,ab OR Sudan*.ti,ab OR Zimbabw*.ti,ab) | (migrant.sh OR exp refugee OR migrant*.ti,ab or refugee*.ti,ab or asylum seeker*.ti,ab or immigrant*.ti,ab or settler*.ti,ab) | (men.ti,ab OR male*.ti,ab OR man*.ti,ab) | (qualitative research.sh OR qualitative*.ti,ab OR focus group*.ti,ab OR interview.sh OR interview*.ti,ab OR thematic analysis.sh OR thematic analys*.ti,ab OR content analysis.sh OR content analys*.ti,ab OR grounded theory*.ti,ab OR personal experience.sh OR personal experience*.ti,ab OR lived experience*.ti,ab OR narrative.sh OR narrative*.ti,ab OR mixed method.ti,ab) |
| **PsycInfo** | exp mental health OR exp mental illness OR exp anxiety OR exp anxiety disorders OR exp Posttraumatic Stress Disorder OR depress*.ti,ab. OR anxiety.ti,ab OR "post traumatic stress disorder".ti,ab. OR ptsd.ti,ab. or trauma*.ti,ab. OR stress*.ti,ab. | exp Help Seeking Behavior OR help seek*.ti,ab OR mental health help seeking.ti,ab OR service use* OR seek advice OR help*ti,ab OR service utilis*.ti,ab OR service utiliz*.ti,ab OR access*.ti,ab | (Africa* OR Egypt* OR Tunisia* OR Algeria* OR Morocc* OR Ghan* OR Mali* OR Nigeria* OR "Democratic Republic of Congo" OR Ethiopia* OR "Cote d'Ivoire*" OR "South Africa*" OR "Burkina Faso" OR Somali* OR "South Sudan*" OR Sudan* OR Zimbabw*).ti,ab. | refugees.sh OR (migrant* OR refugee* OR asylum seeker* OR immigrant* OR settler*).ti,ab | (men OR male* OR man*).ti,ab | (qualitative*.ti,ab. OR exp "qualitative research"/ OR exp "focus group"/ OR "focus group*".ti,ab. OR interview*.ti,ab. OR narrative*.ti,ab. OR "discourse analys*".ti,ab. OR "thematic analys*".ti,ab. OR "content analys*".ti,ab. OR "grounded theory*".ti,ab. OR "lived experience*".ti,ab. OR "personal experience*".ti,ab. OR "mixed method*".ti,ab.) |
| **Web of Sciences** | TS("mental health" OR "mental illness" OR "mental disorder" OR depress* OR anxiety OR "post-traumatic stress disorder" OR ptsd OR trauma* OR stress*) | TS("help seek*" OR "mental health help seek*" OR "service use" OR "seek advice" OR help OR "service utilis*" OR "service utiliz*" OR access*) | TS(Africa* OR Egypt* OR Tunisia* OR Algeria* OR Morocc* OR Ghan* OR Mali* OR Nigeria* OR "Democratic Republic of Congo" OR Ethiopia* OR "Cote d’Ivoire" OR "South Africa*" OR "Burkina Faso" OR Somali* OR "South Sudan*" OR Sudan* OR Zimbabw*) | TS(migrant* OR refugee* OR "asylum seeker*" OR immigrant* OR settler*) | TS(men OR male* OR man*) | TS(qualitative* OR "qualitative research*" OR "focus group*" OR interview* OR narrative* OR "discourse analysis" OR "thematic analys*" OR "content analys*" OR "grounded theory*" OR "lived experience*" OR "personal experience*" OR "mixed method*") |

**Supplementary Table 3. Quality assessments**

| Item | Fauk et al. (2022) | Grupp et al. (2019) | Lechner-Meichsner & Comtesse (2022) | Michlig et al. (2022) | Mölsä et al. (2010) |
| --- | --- | --- | --- | --- | --- |
| 1. Question/objective | ● | ● | ● | ● | ● |
| 1. Study design | ● | ● | ● | ● | ● |
| 1. Context for study | ● | ● | ● | ● | ● |
| 1. Theoretical framework/literature | ● | ● | ● | ● | ● |
| 1. Sampling strategy | ● | ◐ | ◐ | ◐ | ● |
| 1. Data collection methods | ◐ | ◐ | ● | ◐ | ● |
| 1. Data analysis | ● | ● | ● | ● | ◐ |
| 1. Verification procedures | ● | ● | ● | ● | ◯ |
| 1. Conclusions supported | ● | ● | ● | ● | ● |
| 1. Reflexivity | ◯ | ◐ | ◐ | ● | ◐ |
| Overall score | .85 | .85 | .90 | .90 | .80 |

Yes (●), Partial (◐), No (◯)
